# Supplementary material for: Noise resilient exceptional-point voltmeters enabled by oscillation quenching phenomena
Source: Nat Commun. 2023 Sep 7;14:5515. doi: 10.1038/s41467-023-41189-7 (PMC10484910; doi:10.1038/s41467-023-41189-7)
Supplement: Supplementary file 1 — Supplementary Information [file 41467_2023_41189_MOESM1_ESM.pdf]

Supplementary Information:
Noise Resilient Exceptional-Point Voltmeters enabled by Oscillation Quenching Phenomena

Arunn Suntharalingam[1], Lucas Fernández-Alcázar[2,3], Rodion Kononchuk[1], Tsampikos Kottos[1]

[1]Wave Transport in Complex Systems Lab, Department of Physics, Wesleyan University, Middletown CT, USA
[2] Institute for Modeling and Innovative Technology, IMIT (CONICET - UNNE), Corrientes W3404AAS, Argentina
[3] Physics Department, Natural and Exact Science Faculty, Northeastern University of Argentina, Corrientes W3404AAS, Argentina

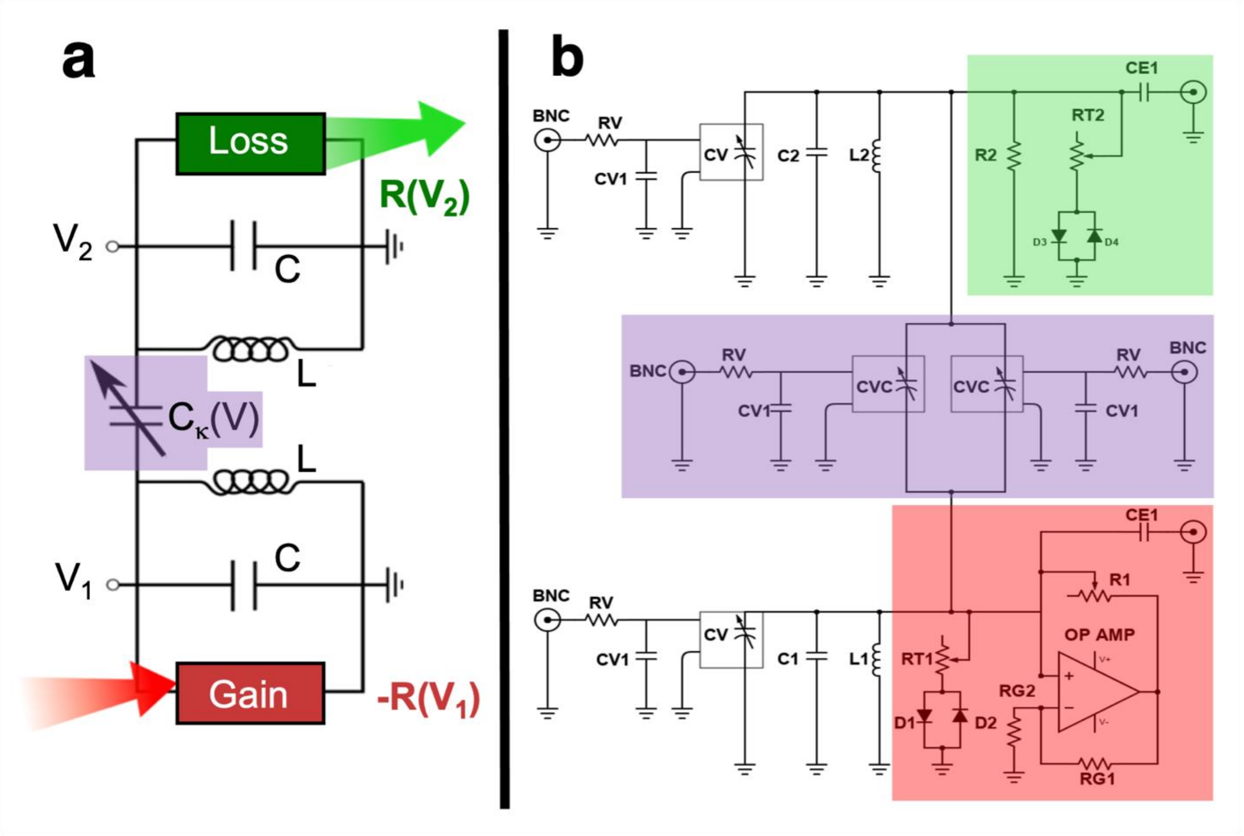

Supplementary Figure 1**: Circuit Schematic. a** The conceptual circuit. **b** The actual circuit diagram. Various parts are highlighted with the same color as in **a**.

Supplementary Note 1: Coupled Mode Theory Modeling of the Electronic Circuit

The nonlinear electronic circuit Supplementary Fig. 1 can be described by the temporal coupled mode theory (TCMT), see Eq. (1) of the main text. Apart from the simplicity and the physical intuition that a TCMT modeling can offer, it also allows us to extrapolate our findings to a broader class of systems described by such TCMT.

We start the derivation of Eq. (1) by expressing the energy stored in the LC resonator as $|\psi|^2$ where the complex modal amplitude of a single LC resonator $\psi(t)$ is

$$\psi(t) = \sqrt{\frac{C}{2}}\left(V(t) - \frac{i}{\omega_0}\dot{V}(t)\right); \qquad \text{(S1)}$$

Above $V(t)$ is the node voltage and $\omega_0 = 2\pi f_0 = \frac{1}{\sqrt{LC}}$ the resonant frequency of the resonator in the absence of dissipation (amplification). Using this representation, we rewrite the circuit equation of the LC resonator, i.e., $\frac{d^2}{dt^2}V + \omega_0^2 V = 0$, as a set of two (uncoupled) first order differential equations i.e., $\frac{d\psi}{dt} = i\omega_0\psi$ and its complex conjugate. The latter relation emphasizes the time dependence of the modal amplitude $\psi \sim e^{i\omega_0 t}$ and $\psi^* \sim e^{-i\omega_0 t}$.

Next, we incorporate in our circuit, an energy dissipation (enhancement) associated with a nonlinear damping (antidamping) channel. We consider nonlinear components that are characterized by the I-V curve $I(V) = \alpha\frac{V}{R^{(0)}} + bV^3$ where $\alpha = 1$ ($\alpha = -1$) represents loss (gain) and $b \approx 7 \cdot 10^{-4} AV^{-3}$. The corresponding Kirchhoff's equation for the individual RLC resonator collapse to the following expression

$$\ddot{V}(t) + \alpha\Gamma^{(0)}\dot{V}(t) + \beta V^2(t)\dot{V}(t) + \omega_0^2 V(t) = 0; \quad \text{(S2)}$$

where $\Gamma^{(0)} = \left(R^{(0)}C\right)^{-1}$ is the relaxation rate of the individual (linear) LRC oscillator, and $\beta = \frac{3b}{C}$. Equation (S2) can be written in terms of ψ and its conjugate ψ^* as

$$2\dot{\psi}^* + 2i\omega_0\psi^* - \left(\alpha\Gamma^{(0)} + \frac{\beta}{2C}|\psi|^2\right)(\psi - \psi^*) - \frac{\beta}{2C}[\psi^3 - (\psi^*)^3] = 0; \qquad \text{(S3)}$$

By invoking a rotating wave approximation and eliminating the fast-oscillating terms, the above equation can be further simplified as

$$i\dot{\psi}^* \approx \omega_0\psi^* - i\frac{\alpha}{2}\Gamma^{(0)}\psi^* - i\chi|\psi^*|^2\psi^*; \qquad \text{(S4)}$$

where $\chi = \frac{\beta}{4C} = \frac{3b}{4C^2}$.

We proceed by developing a TCMT associated to the two coupled RLC tanks. From Kirchhoff's laws we obtain the equations for the voltage V_n in resonator $n = 1,2$

$$\begin{aligned}(1+\kappa)\ddot{V}_1-\kappa\ddot{V}_2-\Gamma_1^{(0)}\dot{V}_1+\beta V_1^2\dot{V}_1+\omega_0^2V_1=0\\(1+\kappa)\ddot{V}_2-\kappa\ddot{V}_1+\Gamma_2^{(0)}\dot{V}_2+\beta V_2^2\dot{V}_2+\omega_0^2V_2=0\end{aligned};\quad \text{(S5)}$$

where $\Gamma_n^{(0)}=\left(R_n^{(0)}C\right)^{-1}$ is the relaxation rate of each resonator (below we will assume the high-Q limit, i.e., $\frac{\Gamma_n^{(0)}}{\omega_0}\ll 1$), $C_\kappa=\kappa C$ is the (voltage controlled) coupling capacitance, with $\kappa\ll 1$ being the coupling strength coefficient. Using the complex-mode representation for each resonator n, we rewrite the circuit equations Eq. (S5) as

$$i\begin{pmatrix}\dot{\psi}_1^*\\\dot{\psi}_2^*\end{pmatrix}=\begin{pmatrix}\omega_0\left(1-\frac{\kappa}{2}\right)+i\frac{\Gamma_1^{(0)}}{2}-i\chi|\psi_1|^2 & \omega_0\frac{\kappa}{2}\\ \omega_0\frac{\kappa}{2} & \omega_0\left(1-\frac{\kappa}{2}\right)-i\frac{\Gamma_2^{(0)}}{2}-i\chi|\psi_2|^2\end{pmatrix}\begin{pmatrix}\psi_1^*\\\psi_2^*\end{pmatrix};\quad \text{(S6)}$$

where we have invoked, as in the case of single RLC resonator, the rotating wave approximation, together with the weak coupling limit $\kappa\ll 1$ and high-Q $\frac{\Gamma_n^{(0)}}{\omega_0}\ll 1$ approximations. We further simplify Eq. (S6) by defining the rescaled complex field $a_n=\sqrt{\frac{\chi}{\omega_0}}\psi_n^*$ and time $\tau=\omega_0 t$,

$$i\frac{d}{d\tau}\begin{pmatrix}a_1\\a_2\end{pmatrix}=\begin{pmatrix}\left(1-\frac{\kappa}{2}\right)+i\left(\gamma_1^{(0)}-|a_1|^2\right) & \frac{\kappa}{2}\\ \frac{\kappa}{2} & \left(1-\frac{\kappa}{2}\right)-i\left(\gamma_2^{(0)}+|a_2|^2\right)\end{pmatrix}\begin{pmatrix}a_1\\a_2\end{pmatrix};\quad \text{(S7)}$$

where $\gamma_n^{(0)}=\frac{\Gamma_n^{(0)}}{2\omega_0}=\frac{1}{2R_n^{(0)}C\omega_0}$.

<u>Supplementary Note 2:</u> Coupling of the Circuit to Transmission Lines

The effect of a weak coupling of the RLC resonators to a transmission line (TL) via a coupling capacitance $C_e=\varepsilon C$, $\varepsilon\ll 1$, can be also modeled using CMT. At the node connecting the TL with the coupling capacitance, the voltage and current flowing toward the n-th ($n=1,2$) RLC resonator can be written as a superposition of forward and backward propagating voltage waves, $V_{TL,n}^{(+)}$ and $V_{TL,n}^{(-)}$

$$V_{TL,n}=V_{TL,n}^{(+)}+V_{TL,n}^{(-)};\quad I_{TL,n}=I_{TL,n}^{(+)}+I_{TL,n}^{(-)}=\frac{1}{z_0}\left(V_{TL,n}^{(+)}-V_{TL,n}^{(-)}\right);\quad \text{(S8)}$$

where $z_0=50\,\Omega$ is the TL's characteristic impedance. In turn, the voltages can be represented by complex wave amplitudes $V_{TL,n}^{(\pm)}=\sqrt{\frac{z_0}{2}}\left(S_n^{(\pm)}+S_n^{(\pm)^*}\right)$, where $S_n^{(\pm)}=$

$\left|S_n^{(\pm)}\right|e^{-i\omega_0 t}$, being $\left|S_n^{(\pm)}\right|$ a slowly varying amplitudes. For a single RLC resonator which is weakly coupled to a TL, we have

$$I_{TL,n} = C_e\left(\dot{V}_{TL,n} - \dot{V}_n\right); \quad -\frac{1}{C}\dot{I}_{TL,n} + \ddot{V}_n + \Gamma_n^{(0)}\dot{V}_n + \omega_0^2 V_n = 0; \quad \text{(S9)}$$

where V_n represents the voltage at the n –th RLC resonator with characteristic frequency ω_0 and decay rate $\Gamma_{\rm n}^{(0)}$. Under the assumptions of impedance matching $\frac{z_0}{Z_0} \sim O(1)$ and weak coupling $\varepsilon \to 0$, we rewrite Eqs. (S8) using the complex mode amplitude of the resonator ψ^* and the input/output wave amplitude $S^{(\pm)}$

$$\begin{gathered} i\frac{d\psi_n^*}{dt} \approx \omega_0\left(1 - \sqrt{\frac{\eta}{2}\frac{Z_0}{z_0}}\right)\psi_n^* - \eta\omega_0\psi_n^* - i\sqrt{2\omega_0\eta}S_n^{(+)} \\ S_n^{(-)} \approx S_n^{(+)} - i\sqrt{2\omega_0\eta}\psi_n^* \end{gathered}; \quad \text{(S10)}$$

where we also invoked the rotating wave approximation, and we introduced the TL-RLC coupling coefficient $\eta \equiv \frac{z_0}{Z_0}\frac{\varepsilon^2}{2}$. Using the transformations $a_n = \sqrt{\frac{\chi}{\omega_0}}\psi_n^*$ and $\tau = \omega_0 t$, and combining Eqs. (S7) and (S10) we arrive to Eq. (1) of the main text which describes the whole system of coupled RLC resonators and TLs. Let us finally point out that when the input wave $S_n^{(+)} = 0$, the output power emitted from the n-th node takes a simple form

$$P_n = \left|S_n^{(-)}\right|^2 \approx 2\omega_0\eta|\psi_n^*|^2 = 2\chi\eta|a_n|^2; \quad \text{(S11)}$$

<u>Supplementary Note 3:</u> Equations of Motion in Polar Form

It is convenient for our analysis, to rewrite Eq. (1) of the main text in polar representation. To this end, we express the complex amplitudes as $a_n(\tau) = A_n(\tau)e^{i\varphi_n(\tau)}e^{-\frac{if\tau}{f_0}}$ where the magnitudes $A_n \geq 0$ and the phases φ_n of the fields in resonator $n = 1,2$ are real numbers. Substitution of these expressions back to Eq. (1) leads to the following set of coupled differential equations

$$\begin{gathered} \dot{A}_1 = \left(\gamma_1^{(0)} - A_1^2 - \eta\right)A_1 + \left(\frac{\kappa}{2}\right)A_2\sin\varphi; \qquad \dot{A}_2 = -\left(\gamma_2^{(0)} + A_2^2 + \eta\right)A_2 - \left(\frac{\kappa}{2}\right)A_1\sin\varphi \\ A_1\dot{\varphi}_1 = \left(\frac{f}{f_0} - \nu_k\right)A_1 - \left(\frac{\kappa}{2}\right)A_2\cos\varphi; \qquad A_2\dot{\varphi}_2 = \left(\frac{f}{f_0} - \nu_k\right)A_2 - \left(\frac{\kappa}{2}\right)A_1\cos\varphi \end{gathered};$$

(S12)

where $\varphi \equiv \varphi_2 - \varphi_1$ is the relative phase. If $A_1, A_2 \neq 0$, the last two equations can be combined in a compact form

$$\dot{\varphi} = \frac{\kappa}{2}\left(\frac{A_2}{A_1} - \frac{A_1}{A_2}\right)\cos\varphi; \qquad \text{(S13)}$$

Finally, in this polar representation the emitted power spectrum Eq. (S10) takes the form $P_n(\omega) = \eta \cdot A_n^2 \left(\frac{4}{3}\frac{1}{bZ_0^2}\right)$.

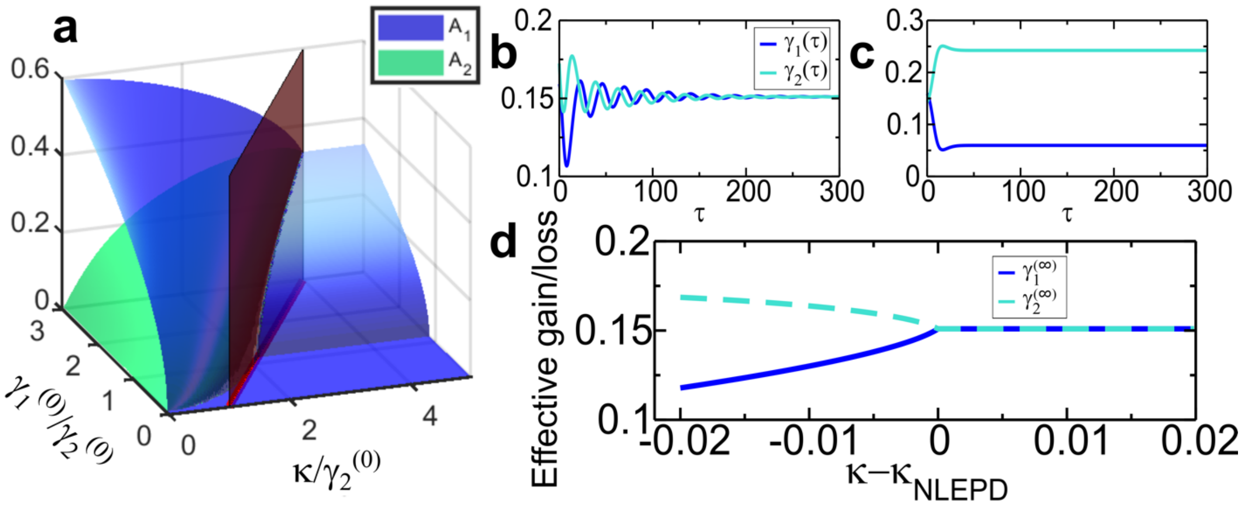

Supplementary Figure 2: **Nonlinear Supermodes and Effective Gain/Loss Coefficient. a** The structure of the nonlinear supermodes (NS) in the $\left(\frac{\kappa}{\gamma_2^{(0)}}, \frac{\gamma_1^{(0)}}{\gamma_2^{(0)}}\right)$ parameter space. Blue (green) surface indicates the value of the field amplitude in the first A_1 (second A_2) RLC resonator. Temporal evolution of the effective gain (loss) coefficients $\gamma_1(\tau)(\gamma_2(\tau))$ for linear gain/loss parameter values $\gamma_1^{(0)} \approx 0.18$, $\gamma_2^{(0)} \approx 0.12$. **b** $\kappa = \kappa_{NLEPD} + 0.1$, in the amplitude death (AD) regime where the system is protected by a parity-time $\mathcal{PT}$-symmetry. **c** $\kappa = \kappa_{NLEPD} - 0.1$, in the oscillation death (OD) regime where the system is explicitly violates the $\mathcal{PT}$-symmetry. **d** Overview of the asymptotic gain/loss coefficients versus the coupling variation from the NLEPD. The coupling to the transmission line (TL) is taken $\eta = 0.01$ while $\kappa_{NLEPD} \approx 0.3$.

<u>Supplementary Note 4:</u> Nonlinear Supermodes

The nonlinear supermodes (NS) correspond to the fixed points of Eqs. (S12, S13). We find the fixed points by requiring constant magnitudes and relative phase, i.e., $\dot{A}_1 = \dot{A}_2 = 0$, and $\dot{\varphi} = 0$. The latter condition leads to three possible scenarios: (1) $A_1 = A_2 \neq 0$, (2) $\cos\varphi = 0$, and (3) a variant of the first case where $A_1 = A_2 = 0$. We refer to this last case as the trivial scenario and it is not relevant for hypersensitive sensing schemes. Therefore, we will not analyze it further. A panorama of the nodal amplitudes (A_1, A_2) is shown in Supplementary Fig. 2a versus the relative gain $\frac{\gamma_1^{(0)}}{\gamma_2^{(0)}}$

and the relative capacitive coupling $\frac{\kappa}{\gamma_2^{(0)}}$. Below, we analyze in detail each one of the first two cases.

Supplementary Note 4a: AD Supermodes

We consider first the case of identical field amplitude in both resonators, i.e., $A_1 = A_2 = A$. This corresponds to the amplitude death (AD) domain, characterized by an exact parity-time ($\mathcal{PT}$) symmetry. From the first set of Eqs. (S12), we find that the field amplitude and the relative phase are given by the expressions

$$A = \sqrt{\frac{\gamma_1^{(0)} - \gamma_2^{(0)} - 2\eta}{2}}; \qquad \sin\varphi = -\frac{\gamma_1^{(0)} + \gamma_2^{(0)}}{\kappa}; \qquad (S14)$$

which, in turn, establishes bounds for the parameters where such a solution exists. From the second of these equations, we conclude that the two supermodes differ in their relative phase, i.e., $\varphi_{\pm} = \left(\frac{-1\pm1}{2}\right)\pi \mp \sin^{-1}\left(\frac{\gamma_1^{(0)}+\gamma_2^{(0)}}{\kappa}\right)$, while the coupling between resonators is bounded by $\kappa \geq \gamma_1^{(0)} + \gamma_2^{(0)}$. This condition determines the boundary between the AD and the oscillation death (OD) domains (regions II and I, respectively in Fig. 1b). On the other hand, the physical requirement that $A \in \mathcal{R}_{>0}$ which guarantees the existence of a non-trivial steady state, leads to the constraint that the gain must be strong enough to overcome the total loss of the system, i.e., $\gamma_1^{(0)} \geq \gamma_2^{(0)} + 2\eta$. This condition determines the boundary between the AD domain and region III (see Fig. 1b of the main text).

The nonlinear eigenfrequencies $f_{\pm}$ associated with the NS of Eq. (S14) can be found by imposing in either of the second set of Eqs. (S12), the fixed-point condition $\dot{\varphi}_{1,2} = 0$ together with the expression for $\cos\varphi_{\pm} = \pm\sqrt{1 - \left(\frac{\gamma_1^{(0)}+\gamma_2^{(0)}}{\kappa}\right)^2}$ derived from the second equation in Eq. (S14). We have for the corresponding frequencies

$$f_{\pm} = f_0 \cdot (\nu_\kappa \pm \frac{1}{2}\sqrt{\kappa^2 - \left(\gamma_1^{(0)} + \gamma_2^{(0)}\right)^2}), \qquad (S15)$$

which indicate the existence of a NLEPD at $\kappa_{NLEPD} = \gamma_1^{(0)} + \gamma_2^{(0)}$ corresponding to the transition between AD and OD domain, where not only the eigenfrequencies but also the eigenvectors coalesce.

Supplementary Note 4b: OD Supermodes

Next, we discuss the fixed points of Eqs. (S12, S13) when $\cos\varphi = 0, i.e. \varphi = \pm\frac{\pi}{2}$. From the first set of Eqs. (S12), we conclude that only the relative phase $\varphi = -\frac{\pi}{2}$ ensures a solution with $A_2^2 \geq 0$. In this case the NS have an asymmetric field amplitude, i.e., $A_1 \neq A_2$ and the corresponding field intensities at each resonator are

$$A_1^2 = \gamma_1^{(0)} - \eta - \frac{\kappa}{2}\rho;\ \ A_2^2 = -\gamma_2^{(0)} - \eta + \frac{\kappa}{2}\rho; \qquad \text{(S16)}$$

where, $\rho = \frac{A_2}{A_1} > 0$ represents the relative field amplitude and is a solution of the following quartic equation

$$0 = 1 - 2\rho\left(\frac{\gamma_2^{(0)}+\eta}{\kappa}\right) - 2\rho^3\left(\frac{\gamma_1^{(0)}-\eta}{\kappa}\right) + \rho^4; \quad \text{(S17)}$$

Out of the four roots of Eq. (S17), one has to select the ones that satisfy (a) $\rho \in \mathcal{R}_{>0}$, and (b) $\rho \leq 2\frac{\left(\gamma_1^{(0)}-\eta\right)}{\kappa}$, such that $A_1^2 \geq 0$, and (c) $\rho \leq \frac{\kappa}{2\left(\gamma_2^{(0)}+\eta\right)}$ such that $A_2^2 \geq 0$ (see Eq. (S16)). It turns out from our extensive numerical analysis that the requirement for the stability of the fixed point in the OD domain, results in $\rho \leq 1$ for the relative field amplitude of the nonlinear supermode Eq. (S16). This output is consistent with the intuition that the intensity at the lossy resonator is smaller than the intensity at the gain resonator at the OD domain where $\mathcal{PT}$-symmetry is explicitly violated.

The corresponding nonlinear eigenfrequency f can be found by substituting in the last Eq. (S12) the value of the relative phase $\varphi = -\frac{\pi}{2}$. In this case, we get

$$f = f_0 \cdot \nu_\kappa; \quad \text{(S18)}$$

Supplementary Note 4c: Symmetry Phases of the Dimer System of Eq. (1)

It is finally instructive to evaluate the effective gain and loss coefficients $\gamma_1(t)$ and $\gamma_2(t)$ as a function of time in the AD and OD regimes. In Supplementary Fig. 2b we show their temporal behavior for a typical set of parameters $\gamma_1^{(0)} \approx 0.18$, $\gamma_2^{(0)} \approx 0.12$ and $\kappa = \kappa_{NLEPD} + 0.1$ for which the system of Eq. (1) is in the AD domain

($\kappa_{NLEPD} \approx 0.3$). We see that in the asymptotic time limit, $\gamma_1^{(\infty)} = \gamma_2^{(\infty)}$, indicating that the electronic dimer is in the exact $\mathcal{PT}$-symmetric phase. Similarly, in Supplementary Fig. 2c we show the temporal behavior of $\gamma_1(t)$ and $\gamma_2(t)$ for the same values of parameters $\gamma_1^{(0)}$, $\gamma_2^{(0)}$ as previously and $\kappa = \kappa_{NLEPD} - 0.1$ for which the system of Eq. (1) is in the OD domain. In this case, the asymptotic values of the effective gain and loss parameters, differ from one-another, i.e., $\gamma_1^{(\infty)} \neq \gamma_2^{(\infty)}$, indicating that the system is in an explicitly broken $\mathcal{PT}$-symmetric phase. A panorama of the asymptotic values $\gamma_1^{(\infty)}, \gamma_2^{(\infty)}$ versus the coupling constant κ for a fixed relative gain $\frac{\gamma_1^{(0)}}{\gamma_2^{(0)}} = 1.5$ is shown in Supplementary Fig. 2d.

<u>Supplementary Note 5:</u> Jacobian Matrix and Stability Analysis in the Parameter Space

The system of Eqs. (S12, S13) can be written in the form $\frac{d\mathbf{u}}{d\tau} = \mathbf{f}(\mathbf{u})$, where $\mathbf{u} = (A_1, A_2, \varphi)^T$. The NS of the previous section $\vec{u}_0$ are obtained by the fixed-point condition $\frac{d\mathbf{u_0}}{d\tau} = \mathbf{f}(\mathbf{u_0}) = 0$. Linearizing the equations of motion around $\mathbf{u_0}$ we get $\mathbf{f}(\mathbf{u_0} + \delta\mathbf{u}) \approx \hat{J}(\mathbf{u_0})\delta\mathbf{u}$, where $\hat{J}(\mathbf{u_0})$ is the Jacobian matrix evaluated at $\mathbf{u_0}$. Subsequently the linearized equations of motion read as,

$$\frac{d\delta\mathbf{u}}{d\tau} = \hat{J}(\mathbf{u_0})\delta\mathbf{u}; \hat{J}(\vec{u}) = \begin{pmatrix} \gamma_1^{(0)} - \eta - 3A_1^2 & \frac{\kappa}{2}\sin\varphi & \frac{\kappa}{2}A_2\cos\varphi \\ -\frac{\kappa}{2}\sin\varphi & \gamma_2^{(0)} - \eta - 3A_2^2 & -\frac{\kappa}{2}A_1\cos\varphi \\ -\frac{\kappa}{2}\cos\varphi\,(\frac{A_2}{A_1^2} + \frac{1}{A_2}) & \frac{\kappa}{2}\cos\varphi\,(\frac{1}{A_1} + \frac{A_1}{A_2^2}) & \frac{\kappa}{2}\sin\varphi\,(\frac{A_1}{A_2} - \frac{A_2}{A_1}) \end{pmatrix}.$$

(S19)

The first Lyapunov criterion allow us to characterize the stability of each nonlinear supermode by analyzing the eigenvalues of the Jacobian matrices evaluated at the NSs $\mathbf{u_0}$. The fixed-point solution $\mathbf{u_0}$ is stable if all the eigenvalues $\{\lambda_n; n = 1,2,3\}$ of the Jacobian (evaluated at the supermode) have negative real part, indicating that the solution is an attractor. If, on the other hand, one of the eigenvalues of the Jacobian has positive real part, the solution $\mathbf{u_0}$ is unstable. In both cases the associated fixed points are classified as hyperbolic equilibria. These equilibria points are further classified as nodes or foci depending on if the corresponding Jacobian eigenvalues are purely real or have also an imaginary component. In case that one

of the eigenvalues of the Jacobian has a zero real part, then the fixed point is characterized as non-hyperbolic. Non-hyperbolic equilibria are not robust to small perturbations (i.e., the system is not structurally stable) and their stability is

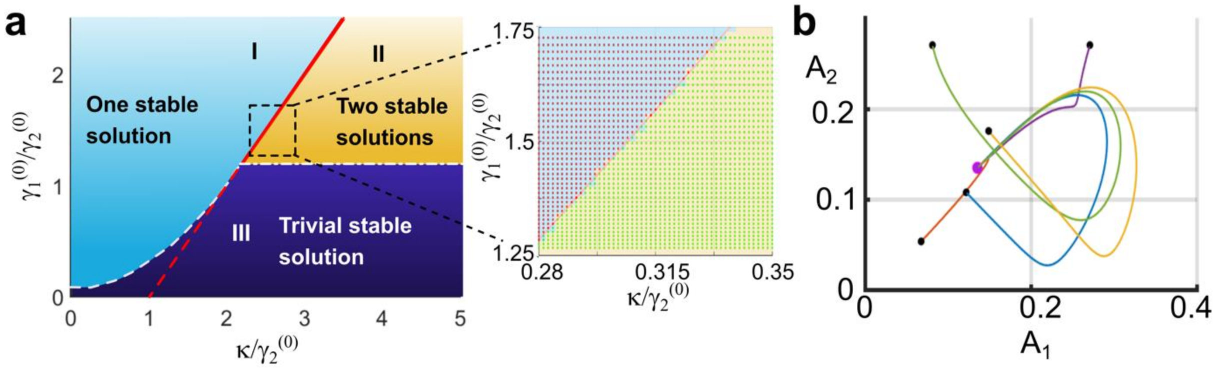

Supplementary Figure 3: **Parameter Space Characterization and Stability of Fixed Points. a** Characterization of the parameter space $(\frac{\kappa}{\gamma_2^{(0)}}, \frac{\gamma_1^{(0)}}{\gamma_2^{(0)}})$ of the nonlinear dimer described by the temporal coupled mode theory (TCMT) Eq. (S12, S13) in terms of the nature and number of its (stable) fixed points. Domain I is identified with the oscillation death (OD) phase and supports one nontrivial stable fixed point; Domain II is identified with the amplitude death (AD) phase and supports two non-trivial stable fixed points; Domain III supports only trivial stable fixed points (i.e., $A_1 = A_2 = 0$). The white dashed lines indicate the borders of various domains (see main text). The red dashed line indicates nonlinear exceptional point degeneracies (NLEPDs) associated with the coalescence of unstable fixed points and occur in the domain III. The red solid line indicates NLEPD associated with the coalescence of stable fixed points and occur at the transition between OD and AD. The inset indicates the parameter domain for which we have analyze the stability (and number) of steady-state solutions of the actual electronic circuit using NGSPICE, a circuit simulator. The blue highlighted area indicates the OD domain while the yellow highlighted area indicates the AD domain. The red line between the two domains matches with the one found from the analysis of the TCMT. **b** Dynamical simulations with Eqs. (S12, S13) with $\kappa = \kappa_{NLEPD}$ indicate that various initial conditions (black circles) in the phase space of the system, converge asymptotically to the corresponding fixed point (violet circle).

determined via direct dynamical simulations with the initial dynamical equations.

A panorama of the $(\kappa, \frac{\gamma_1^{(0)}}{\gamma_2^{(0)}})$ parameter space and its partition to various domains, according to the nature (stable nontrivial versus stable trivial) and number (one, two or none) of stable fixed points, is shown in Supplementary Fig. 3a. This map has been created by analyzing the stability of the TCMT Eq. (1) of the main text (Eqs. (S12, S13) of the Supplementary Information) together with the evaluation of the eigenmodes of the Jacobian matrix Eq. (S19). In the inset of Supplementary Fig.

3a, we show the results of the numerical analysis using NGSPICE. We have simulated the evolution of several initial conditions for various $(\kappa, \frac{\gamma_1^{(0)}}{\gamma_2^{(0)}})$ values in the domain between AD and OD (dashed square) and analyzed the emitted power spectrum associated with the voltage of the gain resonator. The number of extracted frequencies peak(s) and the values of the amplitudes A_1, A_2 have been used to identify the parameter domain.

Let us discuss in more detail the stability of the NLEPD at the transition between AD and OD domains, see Supplementary Fig. 3a. This fixed point turns out to be non-hyperbolic (i.e., one eigenvalue of the Jacobian matrix has zero real part). For an analysis of its stability, we cannot rely on the first Lyapunov criterion. Instead, we have performed direct dynamical simulations for a large ensemble of initial conditions in the phase space of the system and analyze their long-time dynamics. We have found that in all cases, the trajectories are attracted to a final state that is the NLEPD, see Supplementary Fig. 3b.

For completeness of the discussion, we would also like to comment on the unstable non-trivial solutions that exist both in the AD domain and OD domain (see dashed lines in Fig. 2b in the main text). These are fixed points of Eq. (S12) corresponding to $\cos\varphi = 0$ and $(A_1, A_2) \neq (0,0)$ and exists both in the AD and in the OD domains. The analysis of these fixed points follows along the lines of Supplementary Note 4b and correspond to roots of Eq. (S17) with $\rho > 1$. The corresponding frequency of these solutions is given by the either of the second set of Eq. (S12) and are $f_{unstable} = f_0 \cdot \nu_k$.

<u>Supplementary Note 6:</u> Role of Initial Conditions in Determining the Dominating Stable Branch in the AD Domain

In the AD domain, the asymptotic NS results from the choice of the initial conditions $\{A_1(0), A_2(0), \varphi(0)\}$. Figure 2c of the main text suggests that the initial relative phase $\varphi(0)$ determines the evolution of the system towards a specific asymptotic NS. Below, we provide a mathematical argument [32] that shows that the

phases $\varphi(0) = \pm\frac{\pi}{2}$ set the bounds of the basins of attraction and it relates the initial conditions to the NS.

To this end, we consider the projection of the initial wave-vector $|\psi(0)\rangle = (\psi_1, \psi_2)^T$ onto the two NS of the AD domain. Such an operation must be performed carefully, distinguishing left eigenvectors $\langle u_{\pm}| \sim (1, e^{i\varphi_{\pm}})$ from right eigenvectors $|v_{\pm}\rangle \sim \left(1, e^{i\varphi_{\pm}}\right)^T$, which represent the NS (ignoring a multiplicative constant). In such a way, we obtain the complex coefficients $b_{\pm} \equiv \langle u_{\pm}|\psi(0)\rangle$, whose absolute value square is

$$\begin{aligned} |b_+|^2 &= |\psi_1|^2 + |\psi_2|^2 + 2|\psi_1|^2 \cdot |\psi_2|^2 \cdot \cos(\varphi_+ + \varphi(0)) \\ |b_-|^2 &= |\psi_1|^2 + |\psi_2|^2 - 2|\psi_1|^2 \cdot |\psi_2|^2 \cdot \cos(\varphi_+ - \varphi(0)) \end{aligned}; \qquad \text{(S20)}$$

These coefficients determine the NS with larger initial amplitude that, in turn, will dominate the time evolution. From Eq. (S20) one can verify that the boundaries of the basins of attraction correspond to $\varphi(0) = \pm\frac{\pi}{2}$.

<u>Supplementary Note 7:</u> Impact of Resonance Frequency Detuning

Now we consider a small frequency detuning that affects the gain resonator. We describe the field dynamics by an analogous TCMT Eq. (1) of the main text, where we perturb the frequency of the gain resonator, $\nu_\kappa \to \nu_\kappa + \varepsilon$. As we have discussed in detail, for the case $\varepsilon = 0$, there is a bistability around the NLEPD, and different initial conditions let the system evolve to either the f_+ or the f_- NS. The situation is different in the case where a small frequency detuning ε is introduced, with $1 \gg \varepsilon > 0$. Such a detuning smoothens the frequency splitting in the close proximity of the NLEPD without drastically affecting the square-root response of f_+. At the same time, it has important consequences on the stability of the NS in the vicinity of the NLEPD. The effect of a detuning $\varepsilon > 0$ can be appreciated in Supplementary Fig. 4 where we show the frequencies of the NSs. While the upper branch f_+ maintains the stable nature of the associated fixed points, the fixed points associated with the

lower branch f_- become unstable for $\delta V \gtrsim 0$. For large values of δV, the stability of the lower branch is recovered. The stability of the fixed points is determined by analyzing the eigenvalues of the Jacobian, according to the Lyapunov stability criterion. Therefore, in the vicinity of the NLEPD, a small detuning $\varepsilon > 0$ favors the upper branch and renders the lower branch unstable. This explains the fact that our experiment always captures the f_+ fixed point. The exact opposite scenario occurs in case of $\varepsilon < 0$.

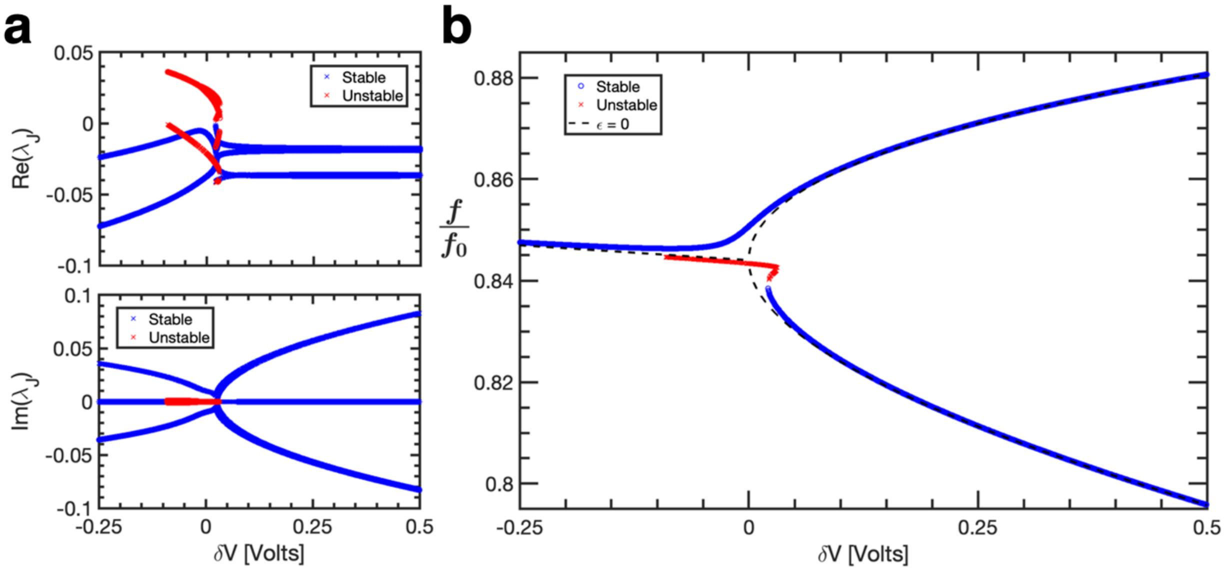

Supplementary Figure 4: **Eigenvalues of the Jacobian and Nonlinear Supermode Frequencies of a Detuned System**. **a** Real and Imaginary part of the eigenvalues of the Jacobian $\{\lambda_n; n = 1,2,3\}$. The blue (red) symbols indicate the stable (unstable) NS. **b** Nonlinear frequencies from the temporal coupled mode theory (TCMT) model. The black dashed line indicates the frequencies in the case of $\varepsilon = 0$.
